# Phytochemical Characterization of *Phoradendron bollanum* and *Viscum album* subs. *austriacum* as Mexican Mistletoe Plants with Antimicrobial Activity

**DOI:** 10.3390/plants10071299

**Published:** 2021-06-26

**Authors:** José Daniel García-García, Julia Cecilia Anguiano-Cabello, Roberto Arredondo-Valdés, Claudio Alexis Candido del Toro, José Luis Martínez-Hernández, Elda Patricia Segura-Ceniceros, Mayela Govea-Salas, Mónica Lizeth González-Chávez, Rodolfo Ramos-González, Sandra Cecilia Esparza-González, Juan Alberto Ascacio-Valdés, Claudia Magdalena López-Badillo, Anna Ilyina

**Affiliations:** 1 Nanobioscience Group, Faculty of Chemistry, Autonomous University of Coahuila, Saltillo 25280, Coahuila, Mexico; daniel_garcia@uadec.edu.mx (J.D.G.-G.); c.candido@uadec.edu.mx (C.A.C.d.T.); jose-martinez@uadec.edu.mx (J.L.M.-H.); psegura@uadec.edu.mx (E.P.S.-C.); m.govea.salas@uadec.edu.mx (M.G.-S.); monicachavez@uadec.edu.mx (M.L.G.-C.); 2 Health Sciences Areas, La Salle Saltillo University, Saltillo 25298, Coahuila, Mexico; julia.anguiano@ulsasaltillo.edu.mx; 3 CONACYT—Faculty of Chemistry Autonomous, University of Coahuila, Saltillo 25280, Coahuila, Mexico; rodolfo.ramos@uadec.edu.mx; 4 Odontology Faculty, Autonomous University of Coahuila, Saltillo 25125, Coahuila, Mexico; sandraesparzagonzal@uadec.edu.mx; 5 Department of Food Research, Faculty of Chemistry, Autonomous University of Coahuila, Saltillo 25280, Coahuila, Mexico; alberto_ascaciovaldes@uadec.edu.mx; 6 Ceramic Materials Academic Group of the Faculty of Chemistry, Autonomous University of Coahuila, Saltillo 25280, Coahuila, Mexico; cllopezb@uadec.edu.mx

**Keywords:** *Phoradendron bollanum*, *Viscum album* subs. *austriacum*, mistletoe, mineral characterization, biochemical characterization, antimicrobial activity

## Abstract

In Mexico, mistletoes have several applications in traditional medicine due to the great variety of compounds with biological activities that have not been characterized to date. The goals of the present study are to analyze the composition of minerals and phytochemical compounds in Mexican mistletoes *Phoradendron bollanum* and *Viscum album* subs. *austriacum* qualitatively and quantitatively, identify the compounds using HPLC-MS, and assess the antimicrobial potential in phytopathogenic microorganism control. Mineral content was evaluated with X-ray fluorescence. Three types of extracts were prepared: ethanol, water, and aqueous 150 mM sodium chloride solution. Characterization was carried out using qualitative tests for phytochemical compound groups, analytical methods for proteins, reducing sugars, total phenol, flavonoids quantification, and HPLC-MS for compound identification. The antimicrobial activity of mistletoe’s liquid extracts was evaluated by microplate assay. K and Ca minerals were observed in both mistletoes. A qualitative test demonstrated alkaloids, carbohydrates, saponins, flavonoids, tannins, and quinones. Ethanolic extract showed flavonoids, 3845 ± 69 and 3067 ± 17.2 mg QE/g for *Phoradendron bollanum* and *Viscum album* subs. *austriacum*, respectively, while aqueous extracts showed a total phenol content of 65 ± 6.9 and 90 ± 1.19 mg GAE/g *Phoradendron bollanum* and *Viscum album* subs. *austriacum*, respectively. HPLC-MS identified largely hydroxycinnamic acids and methoxycinnamic acids. *Clavibacter michiganenses* was successfully inhibited by aqueous extract of both mistletoes.

## 1. Introduction

Mistletoes are hemiparasitic plants that acquire their nutrients by chelating them from the host. These plants are used in traditional medicine to prepare several products such as teas, tinctures, nutritional aspects, and some ointments due to their observed therapeutic effects [1]. In addition to therapeutic use, they are used as ornate plants due to the Nordic tradition. The plant was believed to have magical aspects, as it was related to certain ancient gods. There is even a tradition of kissing under the mistletoe plant to symbolize good luck and fertility [2]. However, it is well known that only a portion of the plant and the proper dosage should be used to prepare tea, as uncontrolled use can cause toxicity and adverse effects on the consumer. For example, compounds such as lectins can cause specific symptoms of inflammation of the gastrointestinal tract [3].

Mistletoes have been studied extensively in Europe and Asia. Many published articles are related to the description of their phytochemical compounds [3], one of the best-known being lectins. Lectins are proteins that can selectively bind to cell-wall carbohydrates. These have been used as adjunctive chemotherapy and radiotherapy treatment in diseases such as cancer [4]. In general, mistletoes share some phytochemicals such as saponin, tannins, and flavonoids. It also depends on the host in which mistletoes grow. Qualitative colorimetric tests also showed alkaloids, cardenolides, and anthraquinones [1].

However, research focused on the description of the properties of Mexican mistletoes is still scarce. Two prominent families of mistletoes are found in Mexico, Central, and South America are *Viscaseae* and *Loranthaceae* [5]. Most of the articles published on mistletoe plants deal with the distribution and identification of the plant genus. The characterization of the phytochemical content provides only an overview of the biological potential of these plants. In Mexico, the National Forestry Commission (CONAFOR) has reported that mistletoe is present in most of the country’s states, including Guerrero, Michoacán, Veracruz, Durango, and Chiapas, among others. The Mexican government has established specific procedures for treating mistletoe-infected forests and green areas [6].

*Phoradendron bollanum* and *Viscum album* subs. *austriacum* have been found in northern and central Mexico [7]. The identification of the phytochemical content of these mistletoe species has not been previously reported. Because the mistletoe is a hemiparasitic plant, which obtains its nutrients from the host, the mineral content can be an exciting point due to this interaction. To date, phytochemical analysis has been performed by qualitative colorimetric tests; however, these tests show a screening of the main compound families present in the plants. HPLC-MS provides a comprehensive analysis of the main components in plant extracts. This technique is useful for the identification, authentication, quantification, and quality control of the composition [5].

The goals of the present study are to characterize the composition of minerals and phytochemical compounds in Mexican mistletoes *P. bollanum* and *V. album* subs. *austriacum* qualitatively and quantitatively, identify the compounds present in extracts obtained with different solvents using HPLC-MS, and assess the potential of the selected extracts in phytopathogenic microorganism control. This is to provide essential preliminary scientific evidence to support and encourage research on Mexican mistletoe plants.

## 2. Results

### 2.1. Elemental Composition

Results of comparative X-ray fluorescence for elemental analysis expressed as a percentage (w/w) in the total amount of ash for *P. bollanum* and *V. album* subs. *austriacum* plants are displayed in Table 1. Mineral contents of *P. bollanum* and *V. album* subs. *austriacum* are different among them. The main mineral components of *V. album* subs. *austriacum* were Ca^2+^ and K^+^, 56.6 and 25.8%, respectively. In *P. bollanum,* these cations are presented at 50.9 and 28.4%, respectively. These minerals are found in both mistletoes; however, the minerals present in the low amounts are part of the main differences. *V. album* subs. *austriacum* is richer in manganese (9.9%) than *P. bollanum*, while *P. bollanum* contains iron and bismuth in higher quantities than *V. album* subs. *austriacum.*, which contains more S, Mg, Fe, P, Sn, and Si than *P. bollanum*. Other minerals were found in trace amounts (Table 1).

### 2.2. Qualitative Phytochemical Analysis of Extracts

Qualitative analysis allows having a general idea of the main groups of compounds present in both mistletoes. Table 2 shows the analysis results for the three extracts of each mistletoe obtained with three different solvents. 

In general, water was the solvent with the most significant number of different compounds extracted. Alkaloids, carbohydrates, flavonoids, coumarins, saponins, tannins, and quinones were found in both extracts of mistletoe. Although ethanol was the solvent with which fewer compounds were extracted, flavones were extracted in high concentrations compared to water and solvent with salt (Table 2). Purines, cyanogenic glycosides, and coumarins with NH_4_OH tests were not detected in the assayed extracts of mistletoe. The results obtained from the qualitative analysis show that both mistletoes have a great variety of bioactive compounds. Although similar compounds were observed in both samples, the intensity of the color was different, which is related to the concentration of each compound. However, it is not enough to affirm the similarity or difference among both mistletoes. This is the reason why spectrophotometric analysis and HPLC-MS were performed.

### 2.3. Protein, Reducing Sugar, and Total Phenol and Flavonoid Contents

Although qualitative analysis has been used for a long time due to its simplicity and speed, it does not provide a concentration of phytochemicals. Quantitative analysis was carried out to determine the concentrations of proteins, reducing sugars, phenols, and total flavonoids. The obtained results are shown in Table 3, which indicates that the concentrations of proteins, reducing sugars, phenols, and flavonoids were higher in extracts of *P. bollanum* than in extracts of *V. album* subs. *austriacum*. In the extraction of proteins and total phenols, the aqueous solvent was the best to obtain higher levels for both mistletoes. The extraction of reducing sugars and flavonoids was favored with the use of ethanol as a solvent. There is no doubt that the assayed mistletoe extracts are rich in flavonoids that can be applied in a wide variety of processes. The results are expressed as milligrams of gallic acid equivalent (mg GAE)/g for total phenol content (TPC) and milligram quercetin equivalent (mg QE)/g for total flavonoid content (TFC).

### 2.4. RP-HPLC-ESI-MS Results

After primary characterization of phytochemical compounds, HPLC-MS was performed. Table 4 shows the obtained results for the mistletoes evaluated: 13 different compounds from 7 different families were found in *P. bollanum* extracts, and 6 compounds from 7 families in *V. album* subsp. *austriacum* extracts. It was possible to observed some compounds extracted with the three solvents assessed. The presence of isomers of (-)-Epicatechin-(2a-7) (4a-8)-epicatechin 3-O-galactoside was observed as well. A greater variety of compounds was found in the extract of *P. bollanum* obtained with water as a solvent. Both mistletoes characterized share similar families such as hydroxycinnamic acids, methoxycinnamic acids, and proanthocyanidin dimers, among others that are discussed further. 

### 2.5. Microbial Inhibition Microplate Assay

**Figure 1:**
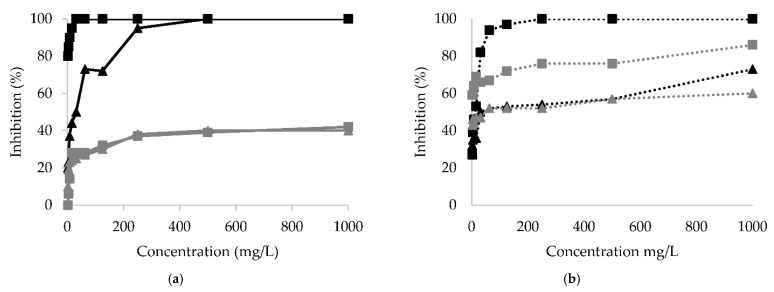
Inhibition percentage for: (**a**) bacteria *Clavibacter michiganensis* (black line) and *Xanthomonas campestris* (gray line) and (**b**) fungus *Alternaria alternata* (black dotted line) and *Fusarium oxysporum* (gray dotted line) with different concentrations of extracts from *P. bollanum* (■) and *V. album* subsp. *austriacum* (▲).

Figure 1 shows the results of *Xanthomonas campestris*, *Clavibacter michiganensis, Alternaria alternata*, and *Fusarium oxysporum* inhibition with different concentrations of aqueous extracts obtained from *P. bollanum* and *V. album* subsp. *austriacum*. These extracts were selected considering various groups of compounds detected in the qualitative assay (Table 2). All phytopathogenic microorganisms were inhibited. The highest inhibitory activity was observed in the case of *Clavibacter michiganensis* with *P. bollanum* extract. 

Table 5 and Figure 1 show that *P. bollanum* extract was the best for inhibiting the tested microorganisms. *Clavibacter michiganensis* and *Alternaria alternata* were the microorganisms most inhibited by the P. bollanum extract. V. album subsp. austriacum extract shows less inhibition activity. However, *Clavibacter michiganensis* was the best inhibited, followed by the fungus Alternaria alternata and Fusarium oxysporum. Xanthomonas campestris was inhibited to a low percentage for both extracts, and the IC_50_ was considerably high.

## 3. Discussion

### 3.1. Elemental Analysis

There are few reports of elemental analysis of mistletoe. So, Mg, Zn, Fe, Cu, and very high concentrations of Ca were reported for African mistletoe [8]. As mentioned, the mistletoe is a hemiparasitic plant, it acquires its nutrients from the host, which causes various damages to the trees. Al-Rowaily studied how mistletoe infection affects the nutritional elements of Acacia trees. They observed that potassium decreased in Acacia trees proportionally to the degree of infection. Compared to uninfected trees, potassium levels decreased by 52% [9].

As a consequence, potassium levels increase in mistletoe plants as the infection progresses. The nutrient content in mistletoes is usually higher than in Acacia trees due to the absorption of elements. The increase in mineral content in mistletoe was also described by Lo Gullo et al. [10]. They assumed that the lack of photosynthetic activity leads to the accumulation of nutritional elements, mainly potassium and calcium.

According to previous reports, the mineral content may vary between mistletoe varieties, while the leaves contain higher amounts of minerals than the branches [11]. The accumulation of mineral elements such as potassium, phosphorus, and sodium in the mistletoe results in low water retention in the host, reducing the plant’s water content [12]. On the other hand, Türe et al. [13] reported that the minerals N, P, K, Na, S, Cu, and Zn are found in higher concentrations in the mistletoe than the host, but the levels of minerals such as Ca, Mg, Fe, and Mn increase in the plant. However, in both samples analyzed in the present study, Ca was higher in concentration than K, which had not been previously reported. This demonstrates the need to evaluate the mineral interaction between Mexican mistletoes and their hosts.

### 3.2. Protein, Reducing Sugar, and Total Phenol and Flavonoid Contents

One of the main applications of mistletoe species is related to the antitumor effect of their extracts. Antitumor activity is probably due to the presence of glycoproteins called lectins, the application of which is considered as an alternative or adjuvant for the treatment of cancer. There are studies with clear evidence of the effect of lectins on cancer cell lines [14]. Thus, *Phoradendron serotinum* showed cytotoxic effects on breast cancer cells, although the observed effect was not attributed to any specific compound. One possible reason may be the presence of lectins in the studied extract [15]. In addition, the *Phoradendron serotinum* extract showed cytotoxic and immunomodulatory effects in vitro against TC-1 cells, while in vivo results demonstrate the stimulation of the immune system to produce cytokines attributed to the components present in *Phoradendron serotinum* [16]. However, to date, we have not found previous reports of lectin isolated from Mexican mistletoe species. European and Asian mistletoe species are considered potential sources of lectins [4]. The present study shows that the extracts of tested Mexican mistletoes have a high amount of proteins. Further studies for the revelation of the presence of lectins among these must be considered.

Table 3 shows the high content of reducing sugars in all analyzed extracts. Results for sugar content may vary depending on host trees. Some studies have reported that inositol and galactose were dominant (58% and 44% by dry weight, respectively) [17]. Although the present work did not intend to identify the sugars present in the sample, their content was demonstrated (Table 3).

Phenol content of the aqueous extract of *P. bollanum* (Table 3) was similar to values reported by Ohikhena for the extract from *Phragmanthera capitata* leaves [18]. Ohikhena also reported that the aqueous solvent was the better solvent for phenol extraction [19]. However, other research demonstrated that acetone and methanol as solvents led to increased phenol extraction [6,7]. Kristiningrum found phenol value lower than 70 mg GAE/g from mistletoe of *Moringa oleifera* lam. (*dendrophthoe pentandra*) with water, ethyl acetate, and n-hexane [20]. This total phenol content was similar to that observed in the present study for *V. album* subs. *austriacum*.

One of the main components observed in this study was flavonoids (Table 3). The amount of flavonoid content from previous reports was not above 679.82 mg QE/g [20]. In this study, 3845 mg QE/g was obtained with ethanol as a solvent. Several flavonoids were found to be soluble in water (Table 3). The presence of flavonoids may be responsible for the great antioxidant capacity that defines the biological activities of mistletoe extracts.

Alharits [21] analyzed the presence of phenols and flavonoids in methanolic extract of leaves and flowers of mistletoe Moringa oleifera. The results showed that the flowers are more affluent than the leaves in phenol and flavonoid content. In addition, some other phytochemical metabolites such as alkaloids, saponins, and terpenoids were observed, as in the present study [21].

### 3.3. RP-HPLC-ESI-MS Results

Hydroxycinnamic acids (HCAs) were the family of compounds most observed in both mistletoes. These compounds were reported as part of some mistletoes in previous investigations [22,23]. Lipid-lowering, antioxidant, antibacterial, and immunomodulatory effects are attributed to HCA, as in the case of *L. cuneifolia* known as “Creole mistletoe” or “Argentine mistletoe” [24]. Coffee is one of the primary sources of HCA. A cup of coffee contains approximately 350 mg of these. Additionally, the wines from grapes are rich in HCA. However, the content may vary depending on the type of grape [25].

HCAs are antioxidant compounds found in the plant cell walls involved in defense against ultraviolet radiation and attacks by pathogens [26]. HCA has shown an anticancer effect against breast, colon, HeLa, and HT-29 cancer cell lines [15,27]. The effect is attributed to the activation of some specific enzymes and, consequently, the inhibition of cell proliferation [28]. Suppression of metastasis, apoptosis, and cell cycle arrest are possible mechanisms by which HCA inhibits proliferation [27]. Yamaguchi [29] found that HCA at a concentration of 1000 nM inhibited the signaling and transcription process and increased the levels of retinoblastoma and regucalcin, which are involved in the suppression of carcinogenesis. Similar observations of cell growth suppression were reported for bone metastatic prostate cancer [29]. *Sida acuta* methanol and water extract were evaluated in breast cancer cells. The IC50 of 102.4 μg/mL was calculated. The main compounds identified by UPLC-MS were hydroxybenzoic and hydroxycinnamic acids [30]. The HCA from mistletoes tested in the present study may likely be a valuable tool in developing treatments for various types of human cancer.

In addition to HCA applications in cancer treatment, these compounds inhibit tyrosinase, the enzyme responsible for the enzymatic browning of fruits and vegetables [31]. Some natural extracts, such as from *Spiranthes sinensis*, are rich in ferulic acid (a compound that belongs to the HCA family) and has been shown to have anti-tyrosinase solid activity [32]. The ethyl acetate fraction of camellia bee pollen showed a content of HCA with anti-tyrosinase activity [33]. Therefore, HCA can inhibit the browning of fruits and vegetables, an essential alternative in agricultural and commercial areas [34].

Methoxycinnamic acids (MCAs), similar to HCAs, are derived from cinnamic acid. These compounds are synthesized as precursors for forming 4-hydroxybenzoic acid, vanillic acid, and syringic acid under normal plant growth conditions [35]. MCA was extracted from mistletoe with ethanolic and aqueous solvents but not with aqueous sodium chloride solution (Table 4).

Tusevski found that MCAs are possibly precursors of derivatives of quinic acid, quercetin, kaempferol, and proanthocyanidin dimers (PCDs) [36]. The PCD was also found in the analyzed extracts and its precursor, 3-feruloylquinic acid, which belongs to the MCA family. MCAs are part of the plant’s metabolism to synthesize different compounds responsible for aromaticity, and some of them have shown antioxidant activities [37]. Their neuroprotective [38] and antihyperglycemic [39] effects have been demonstrated. MCA has been found in coffee pulp [40], in leaves, stems, and flowers *of Miscanthus sinensis* and *Miscanthus sacchariflorus* [41], *Jerez wines* [42], and in *Lonicerae japonicae*, which is a plant used in traditional Chinese medicine [43]. In the extracts analyzed in the present study, MCAs were detected 3-feruloylquinic acid, 4-feruloylquinic acid, and 5-feruloylquinic acid.

In the present study, 4-O-glucoside of gallic acid, which is part of the family of hydroxybenzoic acids (HBAs), was found in an extract from *V. album* subsp. *austriacum* obtained with a liquid solution of NaCl as a solvent. Gallic acid and its derivatives have previously been reported to exhibit anticancer activity in prostate cancer and lymphoblastic leukemia cancer cell lines [20,21].

Flavones are another group of compounds found in the tested extracts (Table 4). These compounds have been widely studied and used in different areas. Flavones are part of the antioxidants that control oxidation processes by eliminating free radicals [44]. Specifically, flavones from Asian mistletoe genera with antimicrobial and hypotensive properties were reported [45]. New flavones from Asian mistletoes, such as lucenin-2, vicenin-2, and stelarin-2, were found [46]. In *V. album* from Romania, hyperoside, isoquercitrin, rutin, and luteolin were detected [47]. Chilean mistletoe leaves showed flavones with high antioxidant activity [48]; for example, sinensetin. This compound, for the first time reported in mistletoe, has antidiabetic properties [49]. However, there is a deficiency in studies focused on the characterization, isolation, and application of mistletoe flavones to date.

Proanthocyanidins are chemical compounds that were found in both mistletoe samples in the present study. These compounds are responsible for plant coloration (red, blue, or purple colors). Hence, the application of these compounds as pigments [50]. In addition, these compounds can help prevent cancer because they are part of flavonoids [51]. Some reports of proanthocyanidin dimers in mistletoe, although the name or identification of these is not specified [52]. In the present study, in both species of mistletoe, (-) - epicatechin- (2a-7) (4a-8) -epicatechin 3-O-galactoside was found, which is known as a proanthocyanidin attenuator. This document may be the first report of the presence of this compound in mistletoes.

Finally, mass spectrometry allowed us to demonstrate and identify catechol and gallocatechin, the compounds present in different plants and recognized as flavonoids [53]. Moustapha reported the presence of (+) - catechin and its derivatives in *P. cuneifolius* and the genus *Psittachathus* [54].

The present study shows that mistletoes *P. bollanum* and *V. album* subsp. *austriacum* have a great variety of bioactive compounds that differ in part from each other.

### 3.4. Microbial Inhibition Microplate Assay

Ohikhena described the antimicrobial activity of mistletoe extracts in bacteria that affect human health [18]. *Acetobacter lwoffii* was inhibited by *Phoradendron serotinum* extract, which is Mexican mistletoe [10]. The methanolic extract of *Korthalsella japonica*, a Korean mistletoe, inhibited *S. epidermidis*, *B. subtilis*, *K. pneumonia* and *E. coli* [46]. Extract from mistletoe *Loranthus micranthus* showed inhibitory effect against *E. coli* and *Proteus vulgaris* [55]. Extract from *Calotropis procera*, an unexplored mistletoe from India growing on mango trees, showed antimicrobial effects against methicillin-resistant bacteria. This allows it to be considered as a possible alternative to existing antibiotics. In the present study, the inhibitory effect of aqueous extracts of *P. bollanum* and *V. album* subsp. *austriacum* against phytopathogenic microorganisms that affect tomato and chili crops was demonstrated.

The obtained findings can significantly impact the development of environmentally clean treatments for the control of phytopathogenic microorganisms.

## 4. Materials and Methods

### 4.1. Sample Collection and Extract Preparation

*P. bollanum* was collected in La Aurora Ecological Park, Saltillo, Coahuila, Mexico (25°26′20.3″ N 100°56′25.2″ W) from Nogales. *V. album* subs. *austriacum* was collected in Orizaba, Veracruz, Mexico (19°10′51.4″ N 96°8.574′ O). The identification of the plants was carried out in the Department of Taxonomy of the Autonomous Agrarian Antonio Narro University, based on the morphological, anatomical, and organoleptic properties of the raw material. The plants (stems and leaves) were washed and dried to a constant weight to eliminate the humidity. Then, it was ground and sieved (≤2 microns). Plant powders were stored in dark bottles until use.

Three types of extracts were prepared and characterized: ethanolic, aqueous, and aqueous with a high concentration of sodium chloride. Ethanolic or aqueous extracts prepared by 14 g of each powdered plant were added to 250 mL of solvent (absolute ethanol or double-distilled water). The mixtures were stirred at 150 rpm for 72 h in the dark at 25 °C. The suspensions were then filtered using Whatman No. 4 filter paper. Ethanol was rotary evaporated while the aqueous extract was lyophilized.

The extract in saline solution was prepared with 10 g of powder from each plant in 100 mL of the NaCl solution at 150 mM. The mixture was stirred at 150 rpm for 1 h at 25 °C, then filtered and lyophilized. The obtained extract powders were stored at four °C in dark bottles until use.

### 4.2. Elemental Composition Characterization

The elemental composition of *P. bollanum* and *V. album* subs. *austriacum* plant powders was estimated by X-ray fluorescence (Equipment Panalytical, Epsilon 1, Almelo (Netherlands)), which is integrated by a spectrometer and uses the Omniam software.

### 4.3. Qualitative Phytochemical Analysis of Extracts

Qualitative analysis was carried out to characterize the groups of phytochemical components of the extracts of *P. bollanum* and *V. album* subs. *austriacum* by standard methods according to the methodology described by Arredondo-Valdés [56]. Six powdered plant extracts (*P. bollanum* and *V. album* subs. *austriacum* with ethanol, water, and water–saline solution NaCl 150 mM each plant) were screened for the presence of alkaloids (with Dragendorff and Sonheschain reagents), carbohydrates (Molisch reagent), carotenoids (with H_2_SO_4_ and FeCl_3_ as reagents), coumarins (Erlich reagent), flavonoids (with Shinoda reagent and 1% NaOH), free reducing sugars (Fehling and Benedict reagents), cyanogenic glycosides (with Grignard reagent), purines (by HCl test), quinones (with NH_4_OH and H_2_SO_4_ reagents for anthraquinones), saponins (by foam test and with Rosenthaler reagent), and tannins (with gelatin, FeCl_3_ and ferrocyanide as reagents).

### 4.4. Protein and Reducing Sugar Content Assays

All extracts (from *P. bollanum* and *V. album* subs. *austriacum*) were prepared at 2000 mg/L in bidistilled water. The Bradford protein assay measured protein. Briefly, 100 μL of the sample was placed in a test tube, and 1 mL of Bradford reagent was added. After 3 min, the absorbance was read at 595 nm in a Thermo Scientific™ Multiskan™ GO microplate spectrophotometer (Waltham, MA, USA) [57]. Protein content was calculated as milligrams of protein per gram of dry weight plant material, using a calibration plot obtained with bovine serum albumin [57]. DNS assay was used to quantify reducing sugars [58]. The reaction was carried out with 1 mL of extract and 2 mL of DNS reagent. After 15 min in a hot water bath (96 °C), 4.5 mL of water was added, and the absorbance was read at 540 nm. A calibration plot was obtained with dextrose.

### 4.5. Total Phenol Content (TPC) Determination

TPC was evaluated by the Folin–Ciocalteu method: 20 μL of the sample (at 2000 mg/L in bidistilled water) was mixed with 120 μL of Na_2_CO_3_ (15% *w*/*v*), 30 μL of Folin–Ciocalteu reagent, and 400 μL of water. The reaction was carried out at 37 °C for 45 min. Thereafter, the absorbance was read at 760 nm on a microplate reader (Thermo Scientific™ Multiskan™ GO, Waltham, MA, USA). The calibration curve was obtained with gallic acid (FagaLab) at concentrations from 100 to 2000 μg/mL. TPC is expressed as milligrams of gallic acid equivalent (mg GAE)/g dry weight plant material [56].

### 4.6. Total Flavonoid Content (TFC) Determination

TFC was quantified by the aluminum chloride method. A 20 μL volume of the extracts (at 2000 mg/L in bidistilled water) was mixed with 0.1 mL of 10% (*w*/*v*) aluminum chloride hexahydrate, 0.1 mL of 1 M potassium acetate, and 2.8 mL of distilled water. After a 40 min incubation period at room temperature, the absorbance was read at 410 nm (Thermo Scientific™ Multiskan™ GO, Waltham, MA, USA). The calibration curve was obtained with quercetin (Sigma-Aldrich Co. Toluca, México) at concentrations from 100 to 2000 μg/mL. TFC was expressed as milligram quercetin equivalent (mg QE)/g dry weight plant material [56].

### 4.7. Analytical RP-HPLC-ESI-MS|

Analyses by reverse-phase–high-performance liquid chromatography were performed on a Varian HPLC system, including an autosampler (Varian ProStar 410, Palo Alto, CA, USA), a ternary pump (Varian ProStar 230I, Palo Alto, CA, USA), and a PDA detector (Varian ProStar 330, USA). A liquid chromatography ion trap mass spectrometer (Varian 500-MS IT Mass Spectrometer, Palo Alto, CA, USA) equipped with an electrospray ion source also was used. Samples (5 µL at 2000 mg/L in bidistilled water and filtered by 0.2 nm cellulose membrane) were injected onto a Denali C18 column (150 mm × 2.1 mm, 3 µm, Grace, Columbia, MD, USA). The oven temperature was maintained at 30 °C. The eluents were formic acid (0.2% *v*/*v*; solvent A) and acetonitrile (solvent B). The following gradient was applied: initial, 3% B; 0–5 min, 9% B linear; 5–15 min, 16% B linear; 15–45 min, 50% B linear. The column was then washed and reconditioned. The flow rate was maintained at 0.2 mL/min, and elution was monitored at 245, 280, 320, and 550 nm. The whole effluent (0.2 mL/min) was injected into the source of the mass spectrometer without splitting. All MS experiments were carried out in the negative mode [M-H] -. Nitrogen was used as nebulizing gas and helium as damping gas. The ion source parameters were spray voltage at 5.0 kV, and capillary voltage and temperature were 90.0 V and 350 °C, respectively. Data were collected and processed using MS Workstation software (V 6.9). Samples were first analyzed in full scan mode in the m/z range 50–2000 [59].

### 4.8. Antimicrobial Activity Microplate Assay

U-well bottom microplates (96-well) were used. Initially, 100 µL of nutritive and Sabouraud broth medium for bacteria and fungus, respectively, was added to all wells. The used microorganisms were *Xanthomonas campestris*, *Clavibacter michiganensis*, *Alternaria alternata*, and *Fusarium oxysporum*. A 40 µL of volume 2, 3, 5-triphenyl tetrazolium chloride (TTC, tetrazolium red, Sigma T-8877, St. Louis, MO, USA.) at 0.01% (*w*/*v*) was added as an indicator, except on the first row of wells. Then, 100 μL of *P. bollanum* or *V. album* subs. *austriacum* aqueous extract at 2 mg/L was added to the four rows of wells and homogenized. Serial dilutions were performed to obtain extract concentrations from 3.9 to 1000 mg/L. Finally, 100 μL of microbial suspension was placed in all bottom wells except on the first row. Bacteria and fungus spores were used at 1 × 10^8^ CFU/mL and spores, respectively. Immediately, microplates were covered and incubated at 28 °C for 48 h. Then, the absorbance was analyzed at 540 nm in the microplate reader (Thermo Scientific™ Multiskan™ GO, Waltham, MA, USA) controlled with Thermo Scientific SkanIt software. The assay was carried out in triplicate for each microorganism. The inhibition percentage was calculated according to the following equation:

% Inhibition = ((Abs control − Abs sample)/(Abs control)) × 100

where Abs control is the absorbance of control without extract, and Abs sample is the absorbance of samples containing extracts. The IC_50_ as the concentration of extracts leads to 50% inhibition, and IC_90_ as the concentration leads to 90% inhibition of microorganism growth were estimated using SAS statistical software [60,61].

## 5. Conclusions

In conclusion, the bioassay found that Mexican mistletoes present several phytochemical compounds and inorganic elements. Here, it was found water is a suitable solvent for the extraction of different biologically active compounds that have antimicrobial activity. Phytochemical content showed mistletoe is rich in biological compounds with potential applications. The composition of the studied mistletoes differs from each other. However, both mistletoes present a high content of flavonoids and phenols. A similar compound was found by HPLC-MS, which can be fractionated for future research in order to evaluate the potential of each compound. The results of the identification of the bioactive compounds by HPLC-MS demonstrated the potential of *P. bollanum* and *V. album* subsp. *austriacum* as carriers of the compounds with diverse activities essential for the health and agricultural sector. The potential of the HPLC-MS technique in plant identification and characterization was demonstrated. Future research focused on the purification of bioactive compounds and their application in the agronomic, health, and biological sectors must be carried out.

## Figures and Tables

**Table 1:** Concentrations of minerals presented *P. bollanum* and *V. album* subsp. *austriacum* quantified by the X-ray fluorescence elemental analysis expressed as a percentage (*w*/*w*) and in parts per million (ppm) in the total amount of ash.

Mistletoe Sample	Element and Content Observed													
	**Mg**	**Al**	**Si**	**P**	**S**	**Cl**	**K**	**Ca**	**Sc**	**Ti**	**V**	**Cr**	**Mn**	**Fe**
*P. bollanum*	NQ	0.98%	2.84%	2.66%	2.71%	0.92%	28.4%	50.9%	0.14%	0.37%	0.01%	0.01%	0.2%	8.11%
*V. album* subsp. *austriacum*	3.257%	0.66%	1.57%	1.7%	1.44%	3.55%	25.8%	56.6%	0.17%	0.17%	0.13%	84 ppm	9.9%	2.92%
	**Co**	**Ni**	**Cu**	**Zn**	**Ga**	**As**	**Se**	**Br**	**Rb**	**Sr**	**Y**	**Zr**	**Nb**	**Mo**
*P. bollanum*	0.08%	0.02%	0.15%	0.59%	32 ppm	65 ppm	11 ppm	0.01%	0.03%	0.5%	56 ppm	0.03%	70 ppm	90 ppm
*V. album* subsp. *austriacum*	0.03%	0.01%	0.11%	0.2%	3 ppm	8 ppm	7 ppm	97 ppm	0.02%	0.08%	34 ppm	64 ppm	35 ppm	79 ppm
	**Tc**	**Ru**	**Rh**	**Pd**	**Cd**	**In**	**Sn**	**Sb**	**Te**	**I**	**Cs**	**Ba**	**Eu**	**Yb**
*P. bollanum*	8 ppm	8 ppm	NQ	47 ppm	58 ppm	10 ppm	0.12%	0.02%	0.04%	19 ppm	0.01%	0.04%	0.05%	0.03%
*V. album* subsp. *austriacum*	5 ppm	17 ppm	3 ppm	3 ppm	40 ppm	6 ppm	2 ppm	69 ppm	0.02%	NQ	0.01%	0.03%	26 ppm	0.02%
	**Hf**	**Ta**	**W**	**Re**	**Os**	**Ir**	**Pt**	**Pb**	**Bi**					
*P. bollanum*	40 ppm	NQ	NQ	32 ppm	NQ	3 ppm	10 ppm	0.03%	10.4%					
*V. album* subsp. *austriacum*	21 ppm	NQ	NQ	5 ppm	NQ	NQ	NQ	43 ppm	13 ppm					
NQ: Not quantified														

**Table 2:** Estimation of phytochemical compound groups of *P. bollanum* and *V. album* subsp. *austriacum*.

Phytochemical Assay	*P. bollanum*			*V. album* subsp. *austriacum*		
	H_2_O + NaCl	H_2_O	EtOH	H_2_O + NaCl	H_2_O	EtOH
Alkaloids:						
by Drangendorff reagent test	+	+	+	++	++	++
by Sonneshein reagent test	+	+	+	+	+	+
Carbohydrates:						
by Molisch reagent test	++	++	+	+++	++	++
Flavonoids:						
by Shinoda reagent test	++	++	+++	++	++	+++
Flavones *	++	++	++	+	+	+
Cyanogenic glycosides	-	-	-	-	-	-
Coumarins:						
with Erlich test	+	+	-	+	+	-
with NH_4_OH test	-	-	-	-	-	-
Reducing sugars:						
by Fehling test	+	+++	++	+	+++	++
by Benedict test	+	+	-	+	+	-
Saponins	+	++	-	+	++	-
Rosenthaler	-	-	+	-	-	+
Tannins:						
by Gelatin test	++	+++	+	++	++	+
by FeCl_3_ ** test	+	++	-	+	++	
by Ferricyanide	-	+	++	-	+	++
Purines	-	-	-	-	-	-
Quinones ***:						
with NH_4_OH	+	+	-	+	+	-
with H_2_SO_4_	+	+	+	+	+	-

* Yellow color = xanthones and flavones; ** Black color = gallic acid derivatives; *** Anthraquinones. Signs are + as lower intensities of observed response; ++ as median intensities of observed response; +++ as higher intensities of the observed response.

**Table 3:** Concentrations of biochemical components of *P. bollanum* and *V. album* subsp. *austriacum* in extracts obtained with different solvents (standard deviations are shown).

Mistletoe	Proteins (mg g^−1^)	Reducing Sugars (mg g^−1^)	Total Phenols (mg GAE/g)	Total Flavonoids (mg QE/g)
***P. bollanum***				
H_2_O + NaCl	12 ± 0.33 ^a^	175 ± 0.73 ^a^	105 ± 5.55 ^a^	537 ± 22 ^a^
H_2_O	36.5 ± 1.2 ^b^	237 ± 4.78 ^b^	165 ± 6.9 ^b^	1110 ± 70 ^b^
EtOH	26.5 ± 0.99 ^c^	336 ± 23.62 ^c^	82 ± 20.28 ^c^	3845 ± 69 ^c^
***V. album* subsp. *austriacum***				
H_2_O + NaCl	17.5 ± 1.1 ^a^	226 ± 0.47 ^a^	68 ± 4.18 ^a^	430 ± 14.5 ^a^
H_2_O	27.5 ± 1.0 ^b^	177 ± 1.87 ^b^	90 ± 1.19 ^b^	725 ± 32.2 ^b^
EtOH	17.5 ± 0.77 ^c^	385 ± 10.62 ^c^	39 ± 6.88 ^c^	3067 ± 17.2 ^c^

Different letters indicate significant differences (*p* < 0.05; ANOVA and Tukey’s HSD test).

**Table 4:** Results of HPLC-MS analysis to identify compounds present in extracts of *P. bollanum* and *V. album* subsp. *austriacum* obtained with ethanol, water, and liquid salt solution.

Solvent	Retention Time (min)	(M-H)-	Compound Identified	Group
***P. bollanum***				
Ethanol	4.32	341	Caffeic acid 4-O-glucoside	Hydroxycinnamic acids
Ethanol	18.56	353	1-Caffeoylquinic acid	Hydroxycinnamic acids
Ethanol	21.696	352.9	3-Caffeoylquinic acid	Hydroxycinnamic acids
Ethanol	25.023	352.9	4-Caffeoylquinic acid	Hydroxycinnamic acids
Ethanol	27.227	367	3-Feruloylquinic acid	Methoxycinnamic acids
Ethanol	31.46	431.1	Apigenin 6-C-glucoside	Flavones
H_2_O	17.354	353	1-Caffeoylquinic acid	Hydroxycinnamic acids
H_2_O	19.907	353	3-Caffeoylquinic acid	Hydroxycinnamic acids
H_2_O	21.087	353	4-Caffeoylquinic acid	Hydroxycinnamic acids
H_2_O	21.532	367	3-Feruloylquinic acid	Methoxycinnamic acids
H_2_O	22.673	705	(-)-Epicatechin-(2a-7)(4a-8)-epicatechin 3-O-galactoside	Proanthocyanidin dimers
H_2_O	24.36	704	(-)-Epicatechin-(2a-7)(4a-8)-epicatechin 3-O-galactoside	Proanthocyanidin dimers
H_2_O	26.619	367	4-Feruloylquinic acid	Methoxycinnamic acids
H_2_O	27.316	563.1	Apigenin arabinoside-glucoside	Flavones
H_2_O	27.567	563.1	Apigenin galactoside-arabinoside	Flavones
H_2_O	28.708	367	5-Feruloylquinic acid	Methoxycinnamic acids
H_2_O	29.3	563.1	Apigenin 7-O-apiosyl-glucoside	Flavones
H_2_O	57.65	367.2	5-Feruloylquinic acid	Methoxycinnamic acids
H_2_O + NaCl	15.71	108.9	Catechol	Other polyphenols
H_2_O + NaCl	17.895	352.9	1-Caffeoylquinic acid	Hydroxycinnamic acids
H_2_O + NaCl	20.285	704.9	(-)-Epicatechin-(2a-7) (4a-8)-epicatechin 3-O-galactoside	Proanthocyanidin dimers
H_2_O + NaCl	23.107	705	(-)-Epicatechin-(2a-7) (4a-8)-epicatechin 3-O-galactoside	Proanthocyanidin dimers
H_2_O + NaCl	24.192	367	4-Feruloylquinic acid	Methoxycinnamic acids
***V. album* subsp. *austriacum***				
Ethanol	3.823	665	Luteolin 7-O-(2-apiosyl-6-malonyl)-glucoside	Flavones
Ethanol	55.776	367.2		5-Feruloylquinic acid Methoxycinnamic acids
Ethanol	58.2	295.2	p-Coumaroyl tartaric acid	Hydroxycinnamic acids
H_2_O	3.281	304.8	(+)-Gallocatechin	Catechins
H_2_O	17.317	284.9	Luteolin	Flavones
H_2_O	25.987	371.1	Sinensetin	Methoxyflavones
H_2_O + NaCl	24.98	703	(-)-Epicatechin-(2a-7) (4a-8)-epicatechin 3-O-galactoside	Proanthocyanidin dimers
H_2_O + NaCl	25.563	707.1	(-)-Epicatechin-(2a-7) (4a-8)-epicatechin 3-O-galactoside	Proanthocyanidin dimers
H_2_O + NaCl	25.83	371.1	Sinensetin	Methoxyflavones
H_2_O + NaCl	58.711	331.1	Gallic acid 4-O-glucoside	Hydroxybenzoic acids

**Table 5:** Estimation of concentrations of 50% (IC_50_) and 90% (IC_90_) of microorganism inhibition for aqueous extracts obtained from *P. bollanum* and *V. album* subsp. *austriacum* (standard deviations are shown).

Microorganism	Concentration (µg/mL)	
	50% of Inhibition	90% of Inhibition
***P. bollanum***		
*Xanthomonas campestris*	1178 ± 5.6 ^a^	377,117 ± 110.23 ^a^
*Clavibacter michiganensis*	0.533 ± 0.02 ^c^	5.61 ± 0.66 ^c^
*Alternaria alternata*	7.43 ± 1.02 ^e^	62.90 ± 1.1 ^e^
*Fusarium oxysporum*	0.40 ± 0.03 ^g^	43,915 ± 22.3 ^g^
***V. album subsp. austriacum***		
*Xanthomonas campestris*	3224 ± 10.23 ^b^	25,326,891 ± 220.3 ^b^
*Clavibacter michiganensis*	18.01 ± 1.71 ^d^	234.35 ± 10.88 ^d^
*Alternaria alternata*	64. 80 ± 2.33 ^f^	325,869 ± 25.3 ^f^
*Fusarium oxysporum*	44.24 ± 6.5 ^h^	22.07 ± 2.2 ^h^

Different letters indicate significant differences (*p* < 0.05; ANOVA and Tukey’s HSD test).

## Data Availability

The data presented in this study are available on request from the corresponding author.
